# Sulfide as a soil phytotoxin—a review

**DOI:** 10.3389/fpls.2013.00268

**Published:** 2013-07-22

**Authors:** Leon P. M. Lamers, Laura L. Govers, Inge C. J. M. Janssen, Jeroen J. M. Geurts, Marlies E. W. Van der Welle, Marieke M. Van Katwijk, Tjisse Van der Heide, Jan G. M. Roelofs, Alfons J. P. Smolders

**Affiliations:** ^1^Department of Aquatic Ecology and Environmental Biology, Institute for Water and Wetland Research, Radboud University NijmegenNijmegen, Netherlands; ^2^Department of Environmental Science, Institute for Water and Wetland Research, Radboud University NijmegenNijmegen, Netherlands; ^3^Peel and Maasvallei Regional Water AuthorityVenlo, Netherlands; ^4^B-WARE Research Centre, Radboud University NijmegenNijmegen, Netherlands; ^5^Royal HaskoningDHVRotterdam, Netherlands; ^6^Community and Conservation Ecology Group, Centre for Ecological and Evolutionary Studies, University of GroningenGroningen, Netherlands

**Keywords:** global change, iron, microorganism, oxygen, plant, roots, sulfur, symbiosis

## Abstract

In wetland soils and underwater sediments of marine, brackish and freshwater systems, the strong phytotoxin sulfide may accumulate as a result of microbial reduction of sulfate during anaerobiosis, its level depending on prevailing edaphic conditions. In this review, we compare an extensive body of literature on phytotoxic effects of this reduced sulfur compound in different ecosystem types, and review the effects of sulfide at multiple ecosystem levels: the ecophysiological functioning of individual plants, plant-microbe associations, and community effects including competition and facilitation interactions. Recent publications on multi-species interactions in the rhizosphere show even more complex mechanisms explaining sulfide resistance. It is concluded that sulfide is a potent phytotoxin, profoundly affecting plant fitness and ecosystem functioning in the full range of wetland types including coastal systems, and at several levels. Traditional toxicity testing including hydroponic approaches generally neglect rhizospheric effects, which makes it difficult to extrapolate results to real ecosystem processes. To explain the differential effects of sulfide at the different organizational levels, profound knowledge about the biogeochemical, plant physiological and ecological rhizosphere processes is vital. This information is even more important, as anthropogenic inputs of sulfur into freshwater ecosystems and organic loads into freshwater and marine systems are still much higher than natural levels, and are steeply increasing in Asia. In addition, higher temperatures as a result of global climate change may lead to higher sulfide production rates in shallow waters.

## Introduction: anaerobiosis and soil sulfur transformations

Although sulfur (S) is one of the six macronutrients for plant growth and low availability of S may therefore limit primary production (Marschner, 1995; Leustek and Saito, 1999), the accumulation of reduced sulfur in sediments of aquatic systems and permanent or riparian wetlands (including estuarine and marine) generally causes physiological toxicity stress for the community involved, including its plants, animals and micro-organisms (Bagarinao, 1992). Unlike the first billion years of life on earth, when sulfide oxidation was an integral part of life generating energy, sulfide accumulation has become much less common as a result of biogenic oxygen production, and sulfide has become toxic to many organisms inhabiting the top layer of soils, including plants (Olsen, 2012). Atmospheric oxygen levels started to increase 2.5 billion years before present (BYBP), and reached levels above 15% since 0.6 BYBP. Oscillations in oxygen and reciprocal oscillations in sulfide levels may even have contributed to mass extinctions (Olsen, 2012).

During flooding and waterlogging of wetland soils, hydrogen sulfide (H_2_S) is produced as a metabolic end product by prokaryotes that oxidize organic compounds using sulfate as a terminal electron acceptor. This group of dissimilatory sulfate reducers includes both Bacteria (e.g., *Desulfovibrio*, *Desulfobacter*) and Archaea (e.g., *Archaeoglobus*). If the sulfide produced cannot be sufficiently sequestered in the soil by metals such as iron, free (dissolved) sulfide will accumulate. Sulfide concentrations in sediment porewaters show a large range up to 15 mmol L^−1^ in marine sediments (Bagarinao, 1992). The reduced sulfur compound acts as a potent phytotoxin (equally toxic as cyanide), by inhibiting the activity of cytochrome *c* oxidase in mitochondria, leading to a subsequent blocking of energy production, and by negatively affecting a range of other metal containing enzymes (Koch et al., 1990; Bagarinao, 1992; Raven and Scrimgeour, 1997). The chemical speciation of sulfide (H_2_S, HS^−^ and S^2−^) depends on soil pH (pK1 = 7.2; pK2 = 13.7 for freshwater). Although all forms seem to be equally toxic (Armstrong and Armstrong, 2005), the gaseous H_2_S will normally prevail over both ionic forms in freshwater systems as the pH of most anaerobic soils is buffered around 6–7 as a result of the HCO^−^_3_ - CO_2_ buffering mechanism, resulting in relative H_2_S abundances of 95–60%. In marine systems, however, pH is often around 7.5, leading to a relative abundance of only 30% for H_2_S, and 70% for HS^−^. As a result of the release of acidic compounds and oxygen from roots, pH in the rhizosphere may, however, be lower than in the bulk soil, and the proportion of H_2_S consequently be higher.

In marine and brackish ecosystems, sulfate concentrations are 10 to 1000 times higher compared to freshwater systems (Marschner, 1995), stimulating sulfate reducers that play an imminent role in decomposition (Jørgensen, 1982) and concomitant sulfide production. Hence, the role of sulfide as a potential natural toxin in saline sediments has been well-established (Carlson and Forrest, 1982; Ingold and Havill, 1984; Webb and Mendelssohn, 1996; Raven and Scrimgeour, 1997; Koch and Erskine, 2001; Pedersen et al., 2004). Sulfide toxicity may also occur when levels and inputs of sulfur remain unchanged, but increased loading with organic matter boosts sulfate reduction rates by providing electron donors from its decomposition (Jørgensen, 1982; Armstrong and Armstrong, 2001; Ruiz-Halpern et al., 2008; Van der Heide et al., 2012). In many coastal systems worldwide, organic loading has strongly increased as a result of land use change in the catchment of rivers (Ver et al., 1999). In addition, increased inorganic nutrient loading (from rivers, run-off, urbanization, atmospheric deposition) fuels local organic matter production (e.g., Van Beusekom and De Jonge, 2002). This makes sulfide-related questions here even more urgent than in more pristine areas.

As a result of anthropogenic forcing, plants in freshwater wetlands and aquatic systems are facing much higher concentrations of sulfur at a global scale nowadays (Lamers et al., 1998). The emission of sulfur to the atmosphere and airborne inputs of anthropogenically-derived sulfur into freshwater wetlands have increased considerably over the last decades as a result of extensive mining for fossil fuels and associated combustion (Gorham, 1976; Schindler et al., 1980; Benkovitz et al., 1996; Schlesinger, 1997). Although S deposition has decreased in Europe and North America during the last decade as a result of effective legislation, rates are still much higher than natural background levels, and in Asia, South America and South Africa, S emission and deposition rates are still strongly increasing (Shah et al., 2000; Vallack et al., 2001). Moreover, sulfate loading of groundwater has increased due to aerobic oxidation of deposited sulfide minerals as a result of water table lowering for agriculture (Schuurkes et al., 1988; Heathwaite, 1990; Lamers et al., 1998), and from anaerobic oxidation of reduced sulfur compounds by chemolithoautotrophic coupling of sulfide oxidation and nitrate reduction in nitrate-loaded catchments and wetlands (Haaijer et al., 2006; Burgin and Hamilton, 2008; Smolders et al., 2010). As a result of the discharge of this groundwater and run-off from pastures and shores suffering from drought, surface waters have become richer in sulfate too. The S in terrestrial soil and subsoil originates in part from increased anthropogenic airborne inputs (S legacy), but also from natural marine and estuarine deposits in the Quaternary or in earlier periods. In addition, recent hydrological changes such as increased inputs of riverine water to compensate for water shortage in both agricultural areas and nature reserves (Roelofs, 1991; Smolders and Roelofs, 1993; Lamers et al., 1998), as well as the intrusion of seawater (salinization; Fogli et al., 2002; Chambers and Pederson, 2006) have contributed to increased S inputs into freshwater wetlands.

## Differential sensitivity thresholds for sulfide

Research on sulfide toxicity and physiological stress originally focused on rice (*Oryza sativa*) as a crop plant in relation to acid sulfate soils that accumulate high concentrations of sulfide during anaerobiosis (Okajima and Takagi, 1955; Vámos, 1959; Hollis et al., 1972). Seedlings appeared to be particularly sensitive to sulfide (Joshi et al., 1975). Since then, sulfide toxicity has also been reported for many other wetland species in both freshwater and saline systems, with a wide range of threshold levels for different species.

In Table 1, a literature overview is given for sulfide toxicity of different plant species, grouped by ecosystem type, showing the differential threshold levels and ecophysiological responses to sulfide. As high levels of free sulfide are only present in wetland soils (including aquatic systems), dryland species are not represented in this table. In addition, no data are available on phytotoxic effects for macroalgae and phytoplankton. As phytoplankton only occurs in the photic zone of the water column that contains oxygen, sulfide toxicity is very unlikely to play an important role. Sulfide toxicity will be much less common for macroalgae than for vascular plants, because they only possess rhizoids and do not protrude into the anoxic sediment, but are often attached to substrates such as rock and coral. At low tide, however, sulfide may well accumulate under dense mats of macroalgae. As an example, anoxic conditions and high ammonium levels were measured in *Cladophora* mats, hampering seagrass growth (Hauxwell et al., 2001). Although sulfide was not measured in this study, it can be expected to have led to sulfide accumulation as well, as shown for *Ulva* mats in coastal lakes (Viaroli et al., 1996). Direct effects of sulfide on macroalgae have, as far as we know, not been tested yet. Algal cover can, however, lead to increased sulfide toxicity to seagrasses (Holmer et al., 2011; Thomsen et al., 2012). Sulfide may only accumulate to high concentrations in the surface water if the water is anoxic and its oxidation is prevented, for instance by the cover of floating-leaved vascular plants such as *Eicchornia crassipes*, *Pistia stratiotes*, *Lemna* spp., and floating ferns such as *Salvinia* spp. and *Azolla* spp. Dense layers of these plants effectively block oxygen intrusion from the atmosphere (e.g., Van Kempen et al., 2012). Phytoplankton is lacking in this dark layer, due to photon deficiency.

**Table 1:** **Overview of sulfide toxicity effects reported in marine, brackish and freshwater plants**.

**Ecotype**	**Concentration**	**Observation**	**Method**	**References**
*Species*	**(μmol L^−1^)**			
**SEAGRASS MEADOWS**				
*Halodule wrightii*	2000	AD	Glucose add. to increase SO_4_ red.	Koch et al., 2007
*Posidonia oceanica*	>1800	AD	Glucose add. to increase SO_4_ red.	Frederiksen et al., 2008
*Halophila ovalis*	>150	AP, RP,	Raised T (25–30°C) in aq. exp.	Holmer et al., 2011
*Posidonia oceanica*	1500	AP, AD	Field Fe addition to lower HS^−^	Marbà et al., 2007
*Thalassia testudinum*	>500	AD	Field observation	Borum et al., 2005
*Thalassia testudinum*	5500	AD	Glucose add. to increase SO_4_ red.	Koch et al., 2007
*Thalassia testudinum*	6000	AD (only high T and Sal.)	H_2_S in hydroponic culture	Koch and Erskine, 2001
*Thalassia testudinum*	5000	AD	org. matter to increase SO_4_ red.	Ruiz-Halpern et al., 2008
*Zostera marina*	600/1000	NP (low/high light)	H_2_S inject. microcosm sediment	Goodman et al., 1995
*Zostera marina*	>1800	No indication of AD	Glucose add. to increase SO_4_ red.	Frederiksen et al., 2008
*Zostera noltii*	>200	AP	Omission of *Loripes* bivalves	Van der Heide et al., 2012
*Zostera noltii*	>500	LE (from patches)	org. matter to increase SO_4_ red.	Govers et al. pers. observ.
*Zostera marina*	600	AP, AD	Raised T (18°C) in aq. exp.	Hoffle et al., 2011
**SALT MARSHES**				
*Agrostis stolonifera*	500	AP, NU	H_2_S in hydroponic culture	Van Diggelen et al., 1987
*Halimione portulacoides*	500	AP, NU	H_2_S in hydroponic culture	Van Diggelen et al., 1987
*Salicomia dolichostachya*	>500	AP	H_2_S in hydroponic culture	Van Diggelen et al., 1987
*Salicomia brachystachya*	>500	AP	H_2_S in hydroponic culture	Van Diggelen et al., 1987
*Spartina alterniflora*	1000	AP	Field observation	King et al., 1982
*Spartina alterniflora*	1130	AP, RD	H_2_S in hydroponic culture	Koch and Mendelssohn, 1989
*Spartina alterniflora*	2000–3000	AP, RA, NU	H_2_S in hydroponic culture	Koch et al., 1990
*Spartina alterniflora*	8000	AP	Field observation	Lee, 1999
*Spartina anglica*	500	AP	H_2_S in hydroponic culture	Van Diggelen et al., 1987
**MANGROVES**				
*Avicennia marina* (sl)	500–1000	AP, RP	H_2_S inject. microcosm sediment	McKee, 1993
*Avicennia marina*	>4000	AP	Field observation	McKee, 1993
*Rhizophora mangle* (sl)	>1000	AP	H_2_S inject. microcosm sediment	McKee, 1993
*Rhizophora mangle*	>1000	AP	Field observation	McKee, 1993
**FRESHWATER AQUATIC SYSTEMS**				
*Ceratophyllum demersum*	>500	AP	SO_4_ addition mesocosms	Geurts et al., 2009
*Elodea nutallii*	100	AP	SO_4_ addition enclosures	Van der Welle et al., 2007a
*Elodea nutallii*	150–500	AP	SO_4_ addition mesocosms	Geurts et al., 2009
*Hydrilla verticulata*	100	NP	H_2_S in root hydroponic culture	Wu et al., 2009
*Nitella flexilis*	50	AP	H_2_S injection aquarium sediment	Van der Welle et al., 2006
*Potamogeton compressus*	150–500	AP	SO_4_ addition mesocosms	Geurts et al., 2009
*Statiotes aloides*	10–100	RD	H_2_S in root hydroponic culture	Smolders and Roelofs, 1996
*Stratiotes aloides*	100–600	AP	SO_4_ addition enclosures	Van der Welle et al., 2007a
*Stratiotes aloides*	500	AP	SO_4_ addition mesocosms	Geurts et al., 2009
**FRESH WATER WETLANDS**				
*Calamagrostis epigejos* (sl)	30–50	AP	Natural production in microcosm	Grootjans et al., 1997
*Calla palustris*	150	AP	SO_4_ addition mesocosms	Geurts et al., 2009
*Caltha palustris*	170	AP, Y	H_2_S injection microcosm sed.	Van der Welle et al., 2007b
*Carex disticha*	10–20	AP	SO_4_ addition mesocosms	Lamers et al., 1998
*Carex disticha*	25	LC, RD	H_2_S injection microcosm sed.	Lamers, 2001
*Carex nigra*	10–20	AP	SO_4_ addition mesocosms	Lamers et al., 1998
*Cladium jamaicense*	220/690/920	LE/NP/AD, RD	H_2_S in hydroponic culture	Li et al., 2009
*Equisetum fluviatile*	50/500	AP (unfertilized/fertilized)	SO_4_ addition mesocosms	Geurts et al., 2009
*Juncus acutiflorus*	25/250	RD/AP	H_2_S injection microcosm sed.	Lamers, 2001
*Juncus alpinoarticulatus* (sl)	30–50	AP	Natural production in microcosm	Grootjans et al., 1997
*Juncus effusus*	500	AP	SO_4_ addition mesocosms	Geurts et al., 2009
*Menyanthes trifoliata*	150/>150	AP (unfertilized/fertilized)	SO_4_ addition mesocosms	Geurts et al., 2009
*Menyanthes trifoliata*	>235	AP	Field observation	Armstrong and Boatman, 1967
*Panicum hemitomon*	630	AP, RD	H_2_S in hydroponic culture	Koch and Mendelssohn, 1989
*Panicum hemitomon*	1000	AP, RA, NU	H_2_S in hydroponic culture	Koch et al., 1990
*Phragmites australis*	1400	AD, SR, B	H_2_S in hydroponic culture	Armstrong et al., 1996
*Phragmites australis*	1500	AP	SO_4_ + C addition mesocosms	Howes et al., 2005
*Phragmites australis*	400	AP	Field observation	Chambers, 1997
*Oryza sativa*	170	RP, B, RO, NU(Fe), WU	H_2_S in anaerobic agar	Armstrong and Armstrong, 2005
*Oryza sativa*	160–310	AP	H_2_S in hydroponic culture	Tanaka et al., 1968
*Oryza sativa*	30	AP	H_2_S in hydroponic culture	Hollis et al., 1972
*Oryza sativa* (sl)	10–60	NU (acute), RO	H_2_S in hydroponic culture	Joshi et al., 1975
*Ranunculus lingua*	500	AP	SO_4_ addition mesocosms	Geurts et al., 2009
*Sphagnum cuspidatum*	60	AD	SO_4_ addition mesocosms	Lamers et al., 1999
*Thelypteris palustris*	150	AP	SO_4_ addition mesocosms	Geurts et al., 2009
*Typha domingensis*	920	LE, NP, AD, RD	H_2_S in hydroponic culture	Li et al., 2009

Concentrations are in μmol L^−1^, (sl), seedling. Observations: AP, decreased aboveground productivity; AD, aboveground die-off; B, blockage of gas pathways and vascular blockage; LC, leaf chlorosis; LE, decreased leaf elongation rate; NP, decreased net photosynthetic rate; NU, decreased nutrient uptake; RD, root (and rhizome) die-off; RO, decreased radial oxygen loss; RA, decreased root ADH activity; RP, decreased belowground production; SR, stunted roots; WU, reduced water uptake; Y, decreased photosynthetic yield (PAM fluorescence).

As can be expected in sulfate-rich environments (particularly when they are permanently submerged), seagrass species are relatively tolerant to sulfide (thresholds generally 2000–6000 μmol L^−1^), although negative effects on growth rates have also been reported at levels of 200–500 μmol L^−1^, especially for small species (Table 1). The saltmarsh species *Spartina alterniflora* is also known to survive high concentrations of sulfide up to 8000 μmol L^−1^ (Lee, 1999; Van der Heide, unpubl. results), but lower concentrations may already impair its growth (King et al., 1982). For mangroves, *Rhizophora* seedlings appeared to be more tolerant than those of *Avicennia*, but adult trees of the latter species tolerate much higher concentrations. The high tolerance of saltmarsh and mangrove species makes sense, as they grow on soils that are rich in both organic electron donors (derived from decomposition of the large flux of litter) and the alternative terminal electron acceptor sulfate. This may also suggest that early-successional species (including a number of seagrass species) may be more sensitive to sulfide than late-successional species, as the latter generally live on sites with higher organic matter accumulation in the sediment. In addition, different ecotypes of the same species can be expected to exist due to strong selection, each adapted to their specific habitat.

Most of the larger freshwater helophyte species such as *Phragmites australis* and *Typha domingensis* also show tolerance to relatively high sulfide concentrations (500–1500 μmol L^−1^; Armstrong et al., 1996; Chambers, 1997; Armstrong and Armstrong, 2001; Adema et al., 2003). Sulfur amendment in order to try to control the unbridled expansion of *P. australis* in the USA at the expense of other species, led to sulfide concentrations of 1500 μmol L^−1^, a level that this species demonstrated survival even at higher salinities (Howes et al., 2005).

In contrast, smaller wetland species and aquatic macrophytes show much lower toxicity thresholds between 10 and 250 μmol L^−1^ (Table 1). Some rootless aquatic macrophytes, growing on highly organic soils, such as *Ceratophyllum demersum*, tolerate relatively high concentrations up to 500 μmol L^−1^. *Oryza sativa* shows intermediate levels of tolerance, although the actual level differs among varieties.

## Effects of experimental set-up and different field measurements

Concentrations of dissolved sulfide can be measured colorimetrically, with S^2−^ selective electrodes in immediately fixed and alkalized porewater, *in situ* using micro-electrodes, or by gas chromatography analysis after gas stripping of acidified porewater. In addition, sulfide-selective optodes, which neither need additional reagents nor consume sulfide, have been developed for direct sulfide measurement (Choi, 1998). As sulfide is easily oxidized and correct sulfide measurements depends on accurate pH measurements for a number of methods, the analytical methods used may show differences in accuracy.

The interpretation of results from literature is strongly confounded by the myriad of methods used in the field and in experiments. For field observations, low sulfide concentrations may also indicate high tolerance to microbial sulfide production due to high oxidation rates supported by oxygen supply from roots. For laboratory tests, the experimental set-up may therefore well-interfere with toxicity levels and attendant effects. As we will discuss, the ability or inability of plants to generate an oxidized rhizosphere strongly determines their sensitivity to reduced phytotoxic compounds including ammonium (NH^+^_4_), ferrous iron (Fe^2+^) and H_2_S, (Laan et al., 1989, 1991; Lamers et al., 2012). Therefore, great care has to be taken in the interpretation of hydroponic experiments to the actual effect of the suggested stress conditions under natural conditions. To test the potential toxicity of reduced compounds and separate ecophysiological responses from those related to direct anoxia effects, we therefore stress the importance of an experimental set-up using a realistic substrate in which plants are able to potentially realize a protective rhizospheric environment to cope with both primary (anoxia-related) and secondary (toxicity-related) stress during anaerobiosis. Pezeshki (2001), in his review on wetland plant responses to soil flooding, also pleaded for research differentiating between these effects.

**Figure 1:**
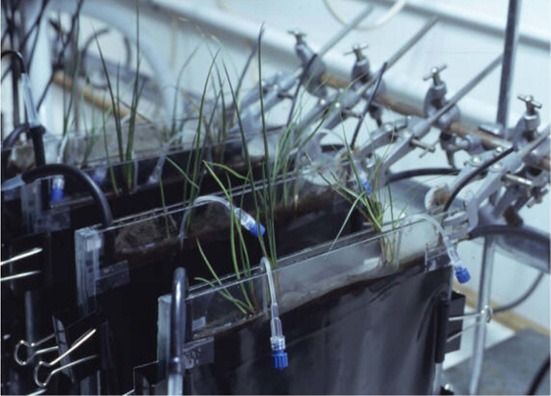
**Example of an experimental set-up using rhizotrons showing inflow, outflow, and the tubes of samplers to collect soil porewater (photo: L. Lamers)**.

On the other hand, the type of soil used in other types of set-up will be very important for the outcome, as this determines the extent of the oxygen sink and diffusion rates. This means that the use of artificial solid substrates, like gels, may also generate experimental artifacts. We therefore suggest using a different approach as an experimental set-up, which includes more realistic edaphic conditions and rhizospheric effects (Figure 1). The actual optimal set-up will depend on the particular questions involved (see, e.g., Van der Heide et al., 2012).

## Sulfur uptake and internal detoxification

Sulfur concentrations in shoots of terrestrial plants are, on average 30 μmol g^−1^ (Gruhlke and Slusarenko, 2012), but values may be higher for freshwater wetland plants (35–150 μmol g^−1^, Van der Welle et al., 2007a,b) and marine plants (100–400 μmol g^−1^; Holmer and Kendrick, 2013), most probably related to the level of S availability in the different environments, but possibly also as a result of the presence of sulfides in the soil. Sulfate is actively taken up by roots and distributed in the plant, with transport through membranes by proton-sulfate co-transporters driven by a proton gradient (Trust and Fry, 1992; Leustek and Saito, 1999). Studies on the abundance ratios of natural S isotopes in *Spartina alterniflora* revealed that most of the sulfate in these marsh plants was derived from sulfide that had partly been oxidized within the plant (Carlson and Forrest, 1982). For seagrasses including *Zostera marina* and *Thalassia testudinum*, isotopic analysis revealed that 50–96% of the S in plants was derived from different sediment sulfides (Frederiksen et al., 2006; Holmer et al., 2009), even when dissolved sulfide concentrations were low (Holmer and Kendrick, 2013). In small seagrass species, sulfur easily enters the roots, and is transported through rhizomes and stems into the leaves, but in taller species its transport seems to be more limited (Holmer and Kendrick, 2013). It is therefore quite probable that in addition to sulfide oxidation in the rhizosphere and subsequent sulfate uptake, gaseous H_2_S is transported to the leaves through the aerenchyma, especially during the night (Pedersen et al., 2004; Holmer and Kendrick, 2013). As the uptake of sulfate after rhizospheric and internal sulfide oxidation generates similar δ^34^S values in plants as direct sulfide uptake, it may be difficult to differentiate between both uptake pathways (Trust and Fry, 1992).

Studies related to S uptake and metabolism have generally been conducted with terrestrial plants species, and differences between sulfide and sulfate uptake and their metabolic pathways are not entirely clear yet. The internal toxicity of sulfide will depend on the species' ability to rapidly metabolize this compound to thiols (organosulfur compound, chemical formula R-SH) such as the amino acids cysteine and methionine and, subsequently, glutathione which is the most abundant thiol in plants (Trust and Fry, 1992; Leustek and Saito, 1999; Hawkesford and De Kok, 2006; Nakamura, 2009). Next, S may be built in a range of different plant tissues. A small number of estuarine plant species, including *Spartina* spp. and *Wollastonia biflora* also produce dimethylsulfoniopropionate (DMSP) from methionine, like a number of marine algal species (Stefels, 2000). This compound may act as a constitutive osmoticum, although its concentration in *Spartina* spp. does not respond to changes in salinity. Alternatively, the production may also provide a mechanism to keep cysteine and methionine levels sufficiently low, and redistribute nitrogen to other amino acids (Stefels, 2000; Otte et al., 2004). In addition, a number of secondary metabolites contain sulfur, including antibiotic substances and odorous compounds (giving flavor to garlic, onions and cabbage) (Leustek and Saito, 1999). The capacity to internally detoxify sulfide is therefore related to cysteine synthesis, catalyzed by the enzyme O-acetylserine(thiol)lyase (OAS-TL) that is present in cytosol, plastids and mitochondria, and an as yet unknown other detoxifying mitochondrial mechanism (Birke et al., 2012). Lee (1999) even hypothesized that low sulfide concentrations might be used by plants to generate energy in mitochondria, similar to the process in microbes and animals. In addition, there is a range of reactive sulfur species next to thiols, such as disulfide-S-oxides (RS(O)_x_SR), sulfenic acids (RSOH), and thiyl radicals (RS) (Gruhlke and Slusarenko, 2012). Although it seems likely that plant hemoglobin (Hb; Igamberdiev et al., 2005) and other metalloproteins may be related to internal sulfide detoxification, similar to Hb in vertebrates and invertebrates (Beauchamp et al., 1984; Weber and Vinogradow, 2001), this is yet to be studied. Next to the metabolic conversion of sulfide, the emission of sulfide from plants, as shown during the exposition to high sulfide concentrations (roots) or SO_2_ concentrations (shoots) (Trust and Fry, 1992), may offer protection. For *Spartina alterniflora* it has been shown that leaves show substantial loss of DMSP during high tide (Pakulski and Kiene, 1992), which provides a mechanism to dissipate excess S. Additionally, the loss of dimethylsulfide (DMS), a volatile metabolite of DMSP, may also offer protection against high S accumulation in a number of estuarine plants (Stefels, 2000).

## Effects on nutrient uptake

Sulfide is known to be able to hamper plant nutrient uptake, which is not surprising given its basic disturbance of cell metabolism and energy transfer. In addition, root loss due to die-off and concomitantly decreased root to shoot ratios lead to an unbalanced nutrient uptake. Sulfide can impair the uptake of nitrogen (N) (Koch et al., 1990), phosphorus (Van der Heide et al., 2012) and Fe (Smolders and Roelofs, 1996; Armstrong and Armstrong, 2005). Depending on the type of nutrient limitation, growth rates may be impaired, while Fe deficiency may lead to lower photosynthetic rates as a result of hampered chlorophyll synthesis. The effects of sulfide on the uptake of Fe and other metals can, however, also be the result of precipitation (Lamers et al., 2012). Although MgS is highly reactive in water and MgSO_4_ is quite soluble, it has been shown that Mg and Ca concentrations in acid sulfate soils are generally undersaturated and governed by cation exchange rather than by their activities. It is well-known that this phenomenon can lead to Mg and Ca deficiency of *Oryza sativa* growing on these soils (Tanaka et al., 1968; Moore and Patrick, 1989). Next, acid production as a result of sulfide oxidation can lead to loss of Mg and Ca from soil cation exchange sites in the rhizosphere, and concomitant lower availability of these macro-ions. For field measurements, however, negative correlations between nutrient uptake and sulfide do not prove sulfide toxicity, as salinity, soil organic matter concentration, and oxygen and nutrient availability are often changing as well along the gradient. Effects of sulfide on soil biogeochemistry affecting plant performance and fitness will be explained further in sections below.

An interesting, but as yet unknown mechanism of sulfide toxicity on plant nutrient uptake might act through its effects of mycorrhizal activity. Although lead sulfides are known to seriously decrease the vitality of ectomycorrhizae (Fomina et al., 2005), the effect of free sulfide on mycorrhizae, and thereby on plant fitness, remains to be elucidated.

## Physico-chemical protection: sequestration in the soil and volatilization

**Figure 2:**
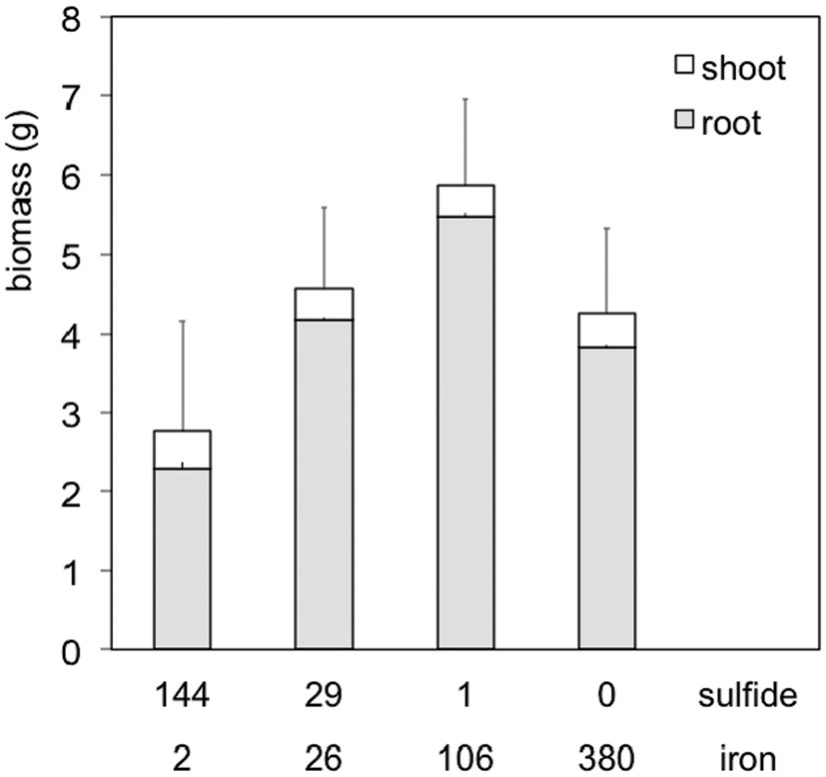
**Interacting effects of dissolved sulfide and iron (concentrations in μmol L^−1^) in the soil porewater on biomass production of *Caltha palustris*.** Although both compounds may be toxic for plant growth, they detoxify one another as a result of FeS_x_ precipitation (quadratic correlation, *p* = 0.014). Adapted from Van der Welle et al. (2006).

Even with high rates of sulfate reduction in the field, the accumulation of dissolved sulfide and its phytotoxic effects can be moderate, or largely absent due to metal sequestration, mainly by Fe. For Fe this leads to the formation of FeS and FeS_2_ (pyrite), detoxifying sulfide (Figure 2; Smolders et al., 1995; Lamers et al., 2002b; Van der Welle et al., 2006, 2007a; Marbà et al., 2007). This mechanism was proposed for *Spartina alterniflora* already in 1982 by King et al., who showed for marshes on the barrier island Sapelo (GA, USA) that in spite of similar sulfate reduction rates, sulfide accumulation showed large variations related to Fe availability. In the same way, discharge of Fe-rich groundwater in wetlands and aquatic systems effectively protects against sulfide toxicity (Lamers et al., 2002a). In marine systems, where sediment Fe concentrations are generally low, the experimental addition of Fe has been shown to counteract sulfide toxicity to seagrass (*Posidonia oceanica*) in a similar way (Holmer et al., 2003, 2005; Marbà et al., 2007; Ruiz-Halpern et al., 2008). However, even if *total* Fe concentrations (i.e., in destruates) in the soil are high, H_2_S accumulation may still occur if the amorphous Fe pool is sulfide-saturated by present or past high S reduction rates. This is clearly indicated by low total Fe:S ratios of the soil. Other metals, including Mn, Zn, Hg, Pb, Cd and Cu, may also precipitate sulfide, but are quantitatively much less important in S biogeochemistry (Bagarinao, 1992). Finally, the accumulation of dissolved sulfide can also be toned down by the activity of microbial communities using nitrate or ferric iron as electron acceptor (Friedrich et al., 2001; see above).

As H_2_S is a gas, not only sequestration in the soil but also volatilization to the atmosphere determines sulfide concentrations in sediments (Bagarinao, 1992). In addition, sulfide can be methylated in organic marine and freshwater sediments, and released as dimethylsulfide and methanethiol into the atmosphere (Lomans et al., 2002).

## Biological protection: radial oxygen loss from roots

Many flooded or waterlogged plants show radial oxygen loss (ROL) from their roots, and the level and pattern of ROL is determined by photosynthetic rate, root architecture and root morphology (Armstrong, 1979; Jackson, 1985; McKee et al., 1988; Laan et al., 1991; Jackson and Armstrong, 1999; Visser et al., 2003; Frederiksen and Glud, 2006; Visser and Bögemann, 2006; Voesenek et al., 2006; Deborde et al., 2008). During nighttime, sulfide intrusion into roots and rhizomes is highest (Borum et al., 2005). Rhizosphere oxidation provides an obvious potential defense mechanism against the toxicity of reduced components such as sulfide (Pitts et al., 1972; Mendelssohn and McKee, 1988; Armstrong et al., 1996; Smolders and Roelofs, 1996; Hemminga, 1998; Armstrong and Armstrong, 2001, 2005; Holmer and Storkholm, 2001; Deborde et al., 2008), provided that soil aerobic microbial respiration and concomitant consumption of oxygen do not counteract this effect.

**Figure 3:**
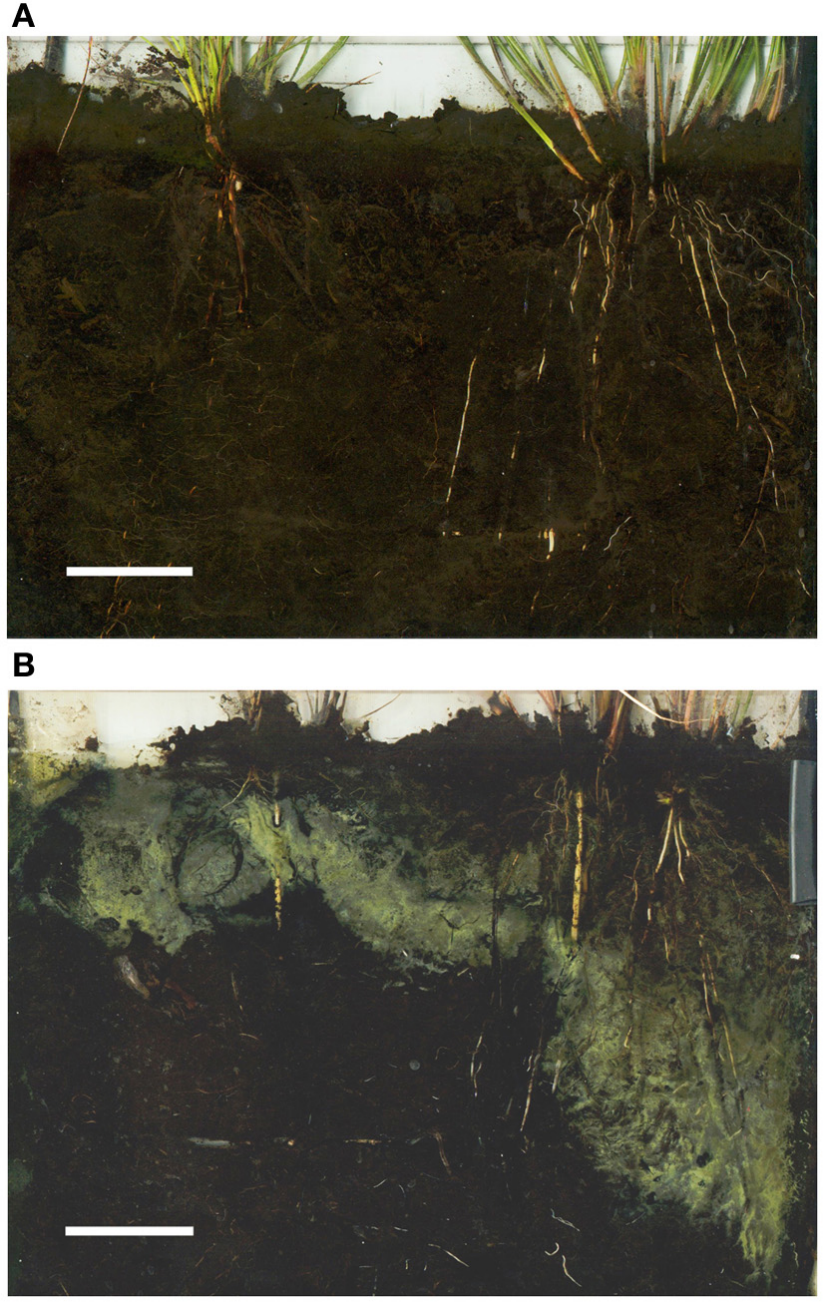
**Rhizotron scans for a control (A) and 250 μmol L^−1^ (B) sulfide treatment (darker soil due to FeS_x_ deposits).** The oxidation potential is clearly visible from the yellow-gray halo of metallic sulfur and other products of oxidation. In each scan, *Carex disticha* is positioned left (only shallow S oxidation halos) and *Juncus acutiflorus* right (deep S oxidation halos). On **(B)**, two white soil moisture samplers are visible. Bars represent 40 mm. Adapted after Lamers (2001) and Lamers et al. (2012).

Spatial differences in oxygen release can not only be attributed to differences in aerenchyma structure, but also to lignine and/or suberine in the epidermis of the roots of different species, preventing loss of all oxygen in the upper soil layer. As an example, the rush species *Juncus acutiflorus* is able to oxidize its rhizosphere, even for the deeper roots, unlike the sedge species *Carex disticha* (Lamers et al., 2012; Figure 3). Although both species did release oxygen from their roots, the relatively high ROL in the top layer solely proved to be insufficient to detoxify sulfide for *C. disticha*, leading to almost complete die-off of deeper roots (Lamers, 2001). In contrast, *J. acutiflorus* was able to completely oxidize its rhizosphere, even in deeper layers where a strong O_2_ demand results from both soil respiration and S oxidation. Observed root loss correlated well with the differences in spatial ROL patterns for both species (Lamers et al., 2012). Sulfide is even known to induce additional suberization (Armstrong and Armstrong, 2005), which can be either an advantage or a disadvantage depending on the location in the roots. This indicates that the specific pattern of ROL, rather than its overall rate, determines the sensitivity of plant species to reduced phytotoxins such as sulfide. For sufficient ROL, the meristematic oxygen content fuelled by photosynthesis during daytime must also be high enough to prevent oxygen depletion by respiration during nighttime. Particularly at higher temperatures, e.g., as a result of climate change in shallow waters, high respiration rates could exceed photosynthetic O_2_ production (Greve et al., 2003).

## Sulfide oxidizers and soil fauna as rhizospheric guards

As H_2_S is readily taken up and causes root toxicity, in contrast to sulfate, the activity of sulfide oxidizing prokaryotes in the rhizosphere is expected to influence both uptake rates and toxicity of S. As the spontaneous chemical oxidation of sulfide is more than 10,000 times slower than biological catalysis (Jørgensen and Revsbech, 1983; Millero, 1986), this rhizosperic sulfur oxidation by prokaryotes (see Friedrich et al., 2001; Ghosh and Roy, 2006) is essential, and the community should comprise large numbers of these organisms living in symbiosis with plants (oxygen supply as a “reward” for detoxification). Sulfur oxidizing microorganisms may be either free living inside or on top of the sediment, in the surface water layer, or associated with roots. Sulfide oxidizers comprise chemolithoautotrophic Proteobacteria such as *Beggiatoa* and *Thiobacillus*, photolithoautotrophic bacteria (e.g., *Rhodovulum, Chromatium*), and chemolitho-autotrophic Archaea (e.g., Sulfolobales) (Ghosh and Dam, 2009). Sulfide-oxidizing prokaryotes may be expected to live inside the root and rhizome aerenchyma as sulfide and oxygen are both present, and *Beggiatoa* presence has indeed been shown inside the rhizomes of seagrass (*Zostera marina*) (Elliott et al., 2006). The oxidation of sulfide in the rhizosphere will, however, also generate acidity in the rhizosphere, slowing down sulfate reduction (Starkey, 1966; Connell and Patrick, 1968) even in the layers beyond the influence of radial oxygen loss by proton diffusion. In this way a second “protective shell” against the adverse effects of sulfate reduction is generated. In addition, the availability of Fe, mobilized by partial FeS_x_ oxidation, may be higher as a result of lower pH values even at a higher redox potential, although a large part will re-precipitate with sulfide. On the other hand, strong acidification of the rhizosphere may also be detrimental to plant roots, e.g., via NH^+^_4_ toxicity (Lucassen et al., 2003; Van den Berg et al., 2005). The outcome of these different rhizospheric processes is determined by the interplay between the rates of ROL, oxygen consumption, sulfide oxidation and acid buffering in the soil.

In addition, next to prokaryotes, a range of eukaryote animal species including invertebrates and fish, have been shown to be able to oxidize sulfide in their mitochondria (whether or not ancient endosymbionts; Gray et al., 1999; Emelyanov, 2003; Olsen, 2012), or by sulfide-oxidizing prokaryotes on internal organs, generating energy (Bagarinao, 1992; Ghosh and Dam, 2009). As sediment bioturbation leads to higher rates of oxygen intrusion, sulfate reduction rates are suppressed even though the availability of readily decomposable organic matter may increase, as was shown for the burrow-forming marine polychaete *Arenicola marina* (lugworm; Nielsen et al., 2003). This not only leads to lower concentrations and toxicity of sulfide, but also to higher availability of Fe^3+^ as an alternative electron acceptor (Nielsen et al., 2003). For this effect, however, the level of bioturbation has to be strong enough to affect rhizospheric sulfide concentrations, especially if organic matter is accumulating in burrows as a result of foraging. Even though fiddler crabs (*Uca* spp.) were able to oxidize the rhizosphere of young mangrove plants (*Laguncularia racemosa*), sulfide levels remained similar (Smith et al., 2009).

**Figure 4:**
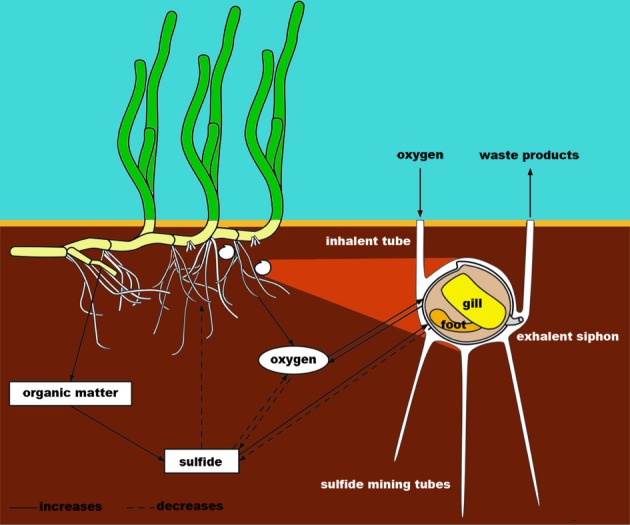
**Sulfide-driven coevolution: tripartite mutualistic interactions among seagrasses, lucinid bivalves and sulfide oxidizing bacteria in their gills generate a higher fitness of all species involved under sulfidic conditions.** See text for explaining mechanisms. Adapted after Van der Heide et al. (2012).

Recently, it was shown in tropical seagrass systems that mutualisms related to rhizospheric S biogeochemistry can be even more complex. Lucinid bivalves containing sulfide-oxidizing symbionts appear to globally occur in tropical and subtropical seagrass meadows (Fisher and Hand, 1984; Van der Heide et al., 2012) and seem strongly associated with these systems ever since seagrasses evolved in the Cretaceous (Van der Heide et al., 2012 and references herein). These lucinids were experimentally shown to play an essential role in seagrass sulfide tolerance, as the sulfide oxidizing prokaryotes living within the gills of the bivalves detoxify sulfide, stimulating seagrass production (Figure 4; Van der Heide et al., 2012). ROL by the seagrass species *Zostera noltii* was only able to reduce the added sulfide concentration from 2700 to 2200 μmol L^−1^, whereas the inclusion of the bivalves led to very low sulfide concentrations of only 15 μmol L^−1^. Simultaneously, the sulfide oxidizers and their host bivalves benefit from the oxygen supplied by ROL from seagrass, and from its organic matter production. It is very likely that similarly elegant mutualistic symbioses involving multiple species have evolved during evolution enabling other plant species to thrive and have higher fitness under sulfidic conditions. We therefore believe that inclusion of plant-symbiont interactions may be a step forward in our ability to explain sulfide tolerance rather than traditional plant physiology alone.

## Indirect toxicity during drought of sulfidic wetlands

Periodic water level fluctuations and severe droughts lead to profound biogeochemical changes in wetlands, due to the strong temporal variation in oxygen concentrations in the soil. Sulfides (free sulfide and metal sulfides) may become toxic in an indirect way in these riparian systems, as the aerobic microbial and chemical oxidation of sulfides generates sulfuric acid (Smolders et al., 2006; Lamers et al., 2012). The actual balance between acid producing and acid consuming processes determines whether this acidification (proton production) leads to an actual drop in pH (increased proton concentration in the porewater). The acid neutralizing capacity (ANC) of soils is determined by the successive extent of bicarbonate and carbonate buffering, the exchange of base cations such as calcium and magnesium at cation binding sites of organic matter and clay, and dissolution of Fe and Al compounds (Scheffer and Schachtschabel, 2002). The total S/(Ca + Mg) ratio of soils may provide an easy proxy to determine the acidification potential of soils during drought (Lucassen et al., 2002). The strong acidification of coastal acid sulfate soils (high concentrations of iron sulfides, low ANC) during droughts, leading to pH values below 4 and concomitant mobilization of aluminum and iron, is a well-known phenomenon, and a problem for rice production and shrimp farming (Dent, 1986; Sammut et al., 1995). Especially in estuarine systems such as marshes, but also in S-rich freshwater systems, massive plant die-off during drought may therefore not only be caused by water deficiency, but also by strong acidification. For the marsh plant *Spartina* spp., it has been shown that the combination of proton toxicity and concomitant mobilization of Al may have contributed to die-off events during droughts (McKee et al., 2004). It has been suggested that acid-tolerant arbuscular mycorrhizal fungi may play an important role in the establishment of pioneer species (grasses, forbs and shrubs) on dry acid sulfate soils (Maki et al., 2008).

## Other biogeochemical processes related to sulfide affecting plant growth

The anthropogenically increased availability of sulfate as an electron acceptor in anaerobic freshwater wetland soils potentially results in eutrophication (Lamers et al., 1998). This is not only caused by increased decomposition and nutrient mineralization rates as a result of the increased availability of sulfate as an electron acceptor, but also by the accumulation of sulfide that lowers phosphate binding to iron oxides and iron hydroxide, thereby increasing phosphate availability in the soil (Ohle, 1954; Sperber, 1958; Caraco et al., 1989; Lamers et al., 1998). Enhanced concentrations of ammonium and phosphate may, however, also result from increased decomposition rates due to greater availability of sulfate as an alternative electron acceptor (Roelofs, 1991; Smolders and Roelofs, 1993; Koerselman et al., 1993; Lamers et al., 1998, 2002b; Zak et al., 2006). For *Thalassia hemprichii*, a seagrass species, it was shown that 80% of its P demand was covered by the activity of sulfate reducers (Holmer et al., 2001). Oxidized sulfur may also be recycled and re-reduced in anaerobic parts of the soil, stimulating decomposition. Under fluctuating oxygen conditions, e.g., in riparian wetlands, reduction and oxidation will therefore alternate (Lucassen et al., 2005).

Sulfate reduction rates can be governed either by the availability of electron donors such as acetate and lactate produced by decomposition of organic matter, or by the availability of sulfate (Lamers et al., 2002b). If, however, high concentrations of a more favorable electron acceptor are available, sulfate-reducing prokaryotes may be partly or completely outcompeted. Wetlands receiving high nitrate loads through discharge of groundwater originating from arable land and fertilized pastures, show low iron and sulfate reduction rates, with concomitantly low phosphate mobilization rates (Lucassen et al., 2004).

In semi-aquatic plants, sulfide toxicity was found to be less pronounced at a higher nutrient availability, possibly as a result of dilution effects by increased growth and increased ROL (Geurts et al., 2009) suggesting that eutrophication may be “masking” sulfide toxicity in polluted areas. For submerged macrophytes, however, eutrophication is expected to aggravate the effects of sulfide, as increased growth of algae and cyanobacteria will directly impair their photosynthetic rates and ability to oxidize the rhizosphere. Filamentous mats of algae on seagrass meadows, resulting from eutrophication, have also been shown to lead to reduced oxygen concentrations in the sediment and increased S uptake and sulfide toxicity in seagrass (Holmer and Nielsen, 2007).

## Sulfide and interspecific interactions: competition, facilitation

**Figure 5:**
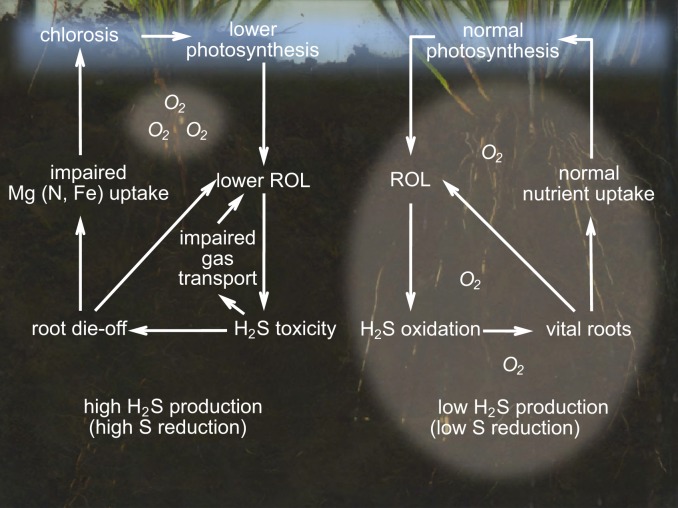
**Conceptual model showing positive feedback loops on plant-rhizophere processes under scenarios of sulfide toxicity (left) and non-toxicity (right).** See text for further explanation. Unlike in other studies, impaired uptake of N or Fe was not found in this study. Impaired gas transport by sulfide has been shown by Armstrong et al. (1996). Sulfide oxidation is carried out by free-living prokaryotes in sediment and water, symbiotic prokaryotes of roots and soil fauna, and mitochondrial metabolism in soil fauna and plants.

Field observations in sulfate-polluted freshwater wetlands suggest that the loss of biodiversity and dominance of a small number of highly competitive plant species may not only be attributed to sulfate-induced eutrophication, but may additionally, or perhaps primarily be triggered by sulfide toxicity (Lamers et al., 2002a). The differential toxicity of hydrogen sulfide provides an additional explanation for changes in competitive strength leading to severe changes in vegetation development in sulfur-loaded wetlands, or in naturally S-rich wetlands that receive higher loads of organic matter. In addition, differences in sulfide accumulation along a gradient may explain vegetation gradients next to salinity effects, e.g., in marshes where *Salicornia* spp. live at the lower, marshes, and high salt marsh species such as *Pucinellia maritime*, *Atriplex patula* and *Festuca rubra* inhabit less sulfidic spots (Ingold and Havill, 1984). Although *Spartina alternifolia* lives at higher marshes than *Salicornia*, the organic content of its sediment is generally higher, potentially leading to higher sulfide accumulation. In freshwater systems, interspecific competition between macrophytes has been shown to depend on the interplay between sulfide and iron in sediments (Van der Welle et al., 2007a). Multiple positive feedback loops therefore increase and stabilize both toxicity and non-toxicity states (Figure 5).

The first constraint on the establishment and competitive strength of wetland plants in sulfur-rich areas, naturally or anthropogenically enhanced, may therefore be sulfide toxicity. As a result, plants that are able to provide their root apices with oxygen without losing all oxygen along the root surface, such as a number of larger graminoids, have a strong competitive advantage, especially if high sulfide oxidation rates are sustained by microbial activity in soils and soil macrofauna. If the growth rate is high, the toxicity effects may be “diluted” and ROL is supported by high photosynthetic rates of the highly competitive species. As a result of these feedbacks, eutrophication and sulfide accumulation in concert may rapidly lead to vegetation changes. From their differential responses to sulfide, Li et al. (2009) argued that the undesirable strong expansion of *Typha* in the Florida Everglades, at the expense of *Cladium*, could partially be explained by the high levels of sulfide (250–375 μmol L^−1^) in this region. These resulted from a combination of high rates of sulfate reduction and low levels of iron to sequester the produced sulfide. For dune slacks it was hypothesized that elevated sulfide concentrations in combination with higher nutrient levels induce a shift to highly productive *Phragmites* stands (Adema et al., 2003). In a recent study on the biogeochemical drivers of species composition in a groundwater-fed freshwater wetland, sulfide appeared to be the most important explaining variable (Simkin et al., 2013). However, in addition to gaining a higher competitive strength, sulfide-detoxifying plant species might also act as ecosystem engineers (sensu Jones et al., 1994) by their facilitation of sulfide-sensitive plants, provided that the latter group is not outcompeted for light by fast-growing species. However, high sulfide levels, in addition to those of other phytotoxins, may have contributed to the large scale *Phragmites* die-back in wetlands loaded with organic compounds (Armstrong and Armstrong, 2001), and to large-scale seagrass die-back events (Carlson et al., 1994; Terrados et al., 1999; Borum et al., 2005). Such massive die-off events have also been shown in constructed wetlands receiving high organic fluxes and showing high sulfide concentrations (above 1000 μmol L^−1^; Wiessner et al., 2008).

## Global effects on wetlands

The risks of sulfide toxicity are an important issue at a global scale, as sulfur concentrations have risen in many freshwater waters and wetlands, including natural vegetation types and rice paddies, due to high anthropogenic S emissions (Smith et al., 2011) and geochemical oxidation processes including the effect of nitrate pollution (Smolders et al., 2010). Although global emissions decreased between 1970 and 2000 due to legislation, they are now increasing significantly again due to the high S emissions of fast-developing regions such as Asia (particularly China) where SO_2_ emissions, as a result of the large-scale use of coal as a fuel, may soon equal the combined emissions of North America and Europe (Shah et al., 2000; Smith et al., 2011). In addition, salinization of coastal freshwater wetlands due to the intrusion of saline groundwater or surface water, and salinization due to the increased frequency of drought episodes in more arid regions increase the risk of sulfide-related vegetation changes during anaerobiosis. To determine the exact causes of salinization on vegetation changes, it is, however, important to experimentally test the effects of sulfide and NaCl separately and in concert. In saline systems (in which sulfate is normally not limiting), increased organic loads will stimulate sulfate reduction rates and lead to higher sulfide levels, especially if temperatures become higher (in shallow waters) as a result of global change (Hoffle et al., 2011; Holmer et al., 2011). Accumulated FeS_x_ in riparian wetlands will massively become oxidized to sulfate during drought (Lucassen et al., 2002), which is prone to renewed reduction during flooding. Even in soils that had not been flooded for more than 10 years, an unexpected diversity of sulfate reducers still appeared to be present and become active after one or two weeks of anaerobiosis (Lamers et al., 1998; Miletto et al., 2008). This shows that the microbial community is very persistent with respect to S biogeochemistry, and able to resuscitate although they have to be classified as “delayed responders” (sensu Placella et al., 2012). As a result, the S legacy of a soil is expected to contribute to sudden die-off of plants in riparian wetlands during anaerobic events.

## General conclusion

As our overview shows that even low concentrations of sulfide are able to 1) affect the ecophysiological functioning individual plants, 2) affect plant competition and facilitation, 3) influence complex rhizospheric mutualisms, and 4) interact with nutrient biogeochemistry, it is clear that sulfide can be a strong driver of ecosystem processes and functioning, also in relation to changing global S balances. Future research should include interactions between plants, microbial communities, soil fauna and soil chemistry, to fully understand and explain differences among plant, vegetation and ecosystem responses to sulfide.

### Conflict of interest statement

The authors declare that the research was conducted in the absence of any commercial or financial relationships that could be construed as a potential conflict of interest.
